# Supplementary material for: Integrative Multi-Omics Analyses Reveal Mechanisms of Resistance to Hsp90β-Selective Inhibition
Source: Cancers (Basel). 2025 Oct 30;17(21):3488. doi: 10.3390/cancers17213488 (PMC12608035; doi:10.3390/cancers17213488)
Supplement: Supplementary file 1 [file cancers-17-03488-s001.zip › Document S1.pdf]

SUPPLEMENTAL FIGURES:

Supplemental material related to Figure 1. Identification of HSP90AB1-dependent and -resistant cancer cell populations.

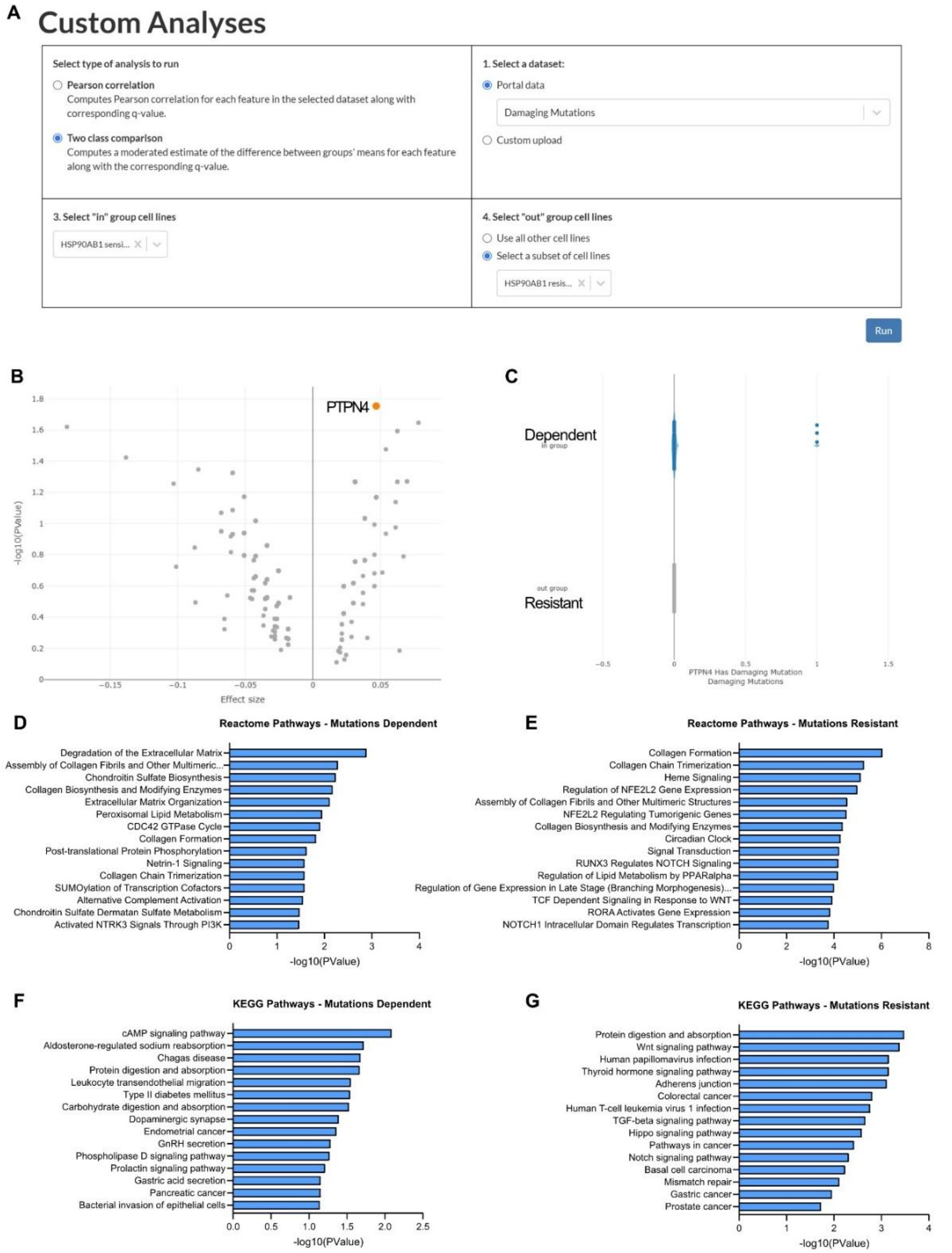

Figure S1. (A–C) Overview of the two-class comparison strategy used to identify features enriched in either dependent or resistant lines. Example analysis setup (A) and volcano plot of results (B) comparing damaging mutations in sensitive and resistant lines and the distribution of cell lines with damaging mutations in PTPN4 gene (C). (D–G) Functional enrichment of group-specific damaging mutations in Reactome and KEGG pathways for each group.

Supplemental material related to Figure 2. Gene expression and metabolite profiling reveal distinct metabolic and signaling programs in HSP90AB1-dependent and -resistant cells.

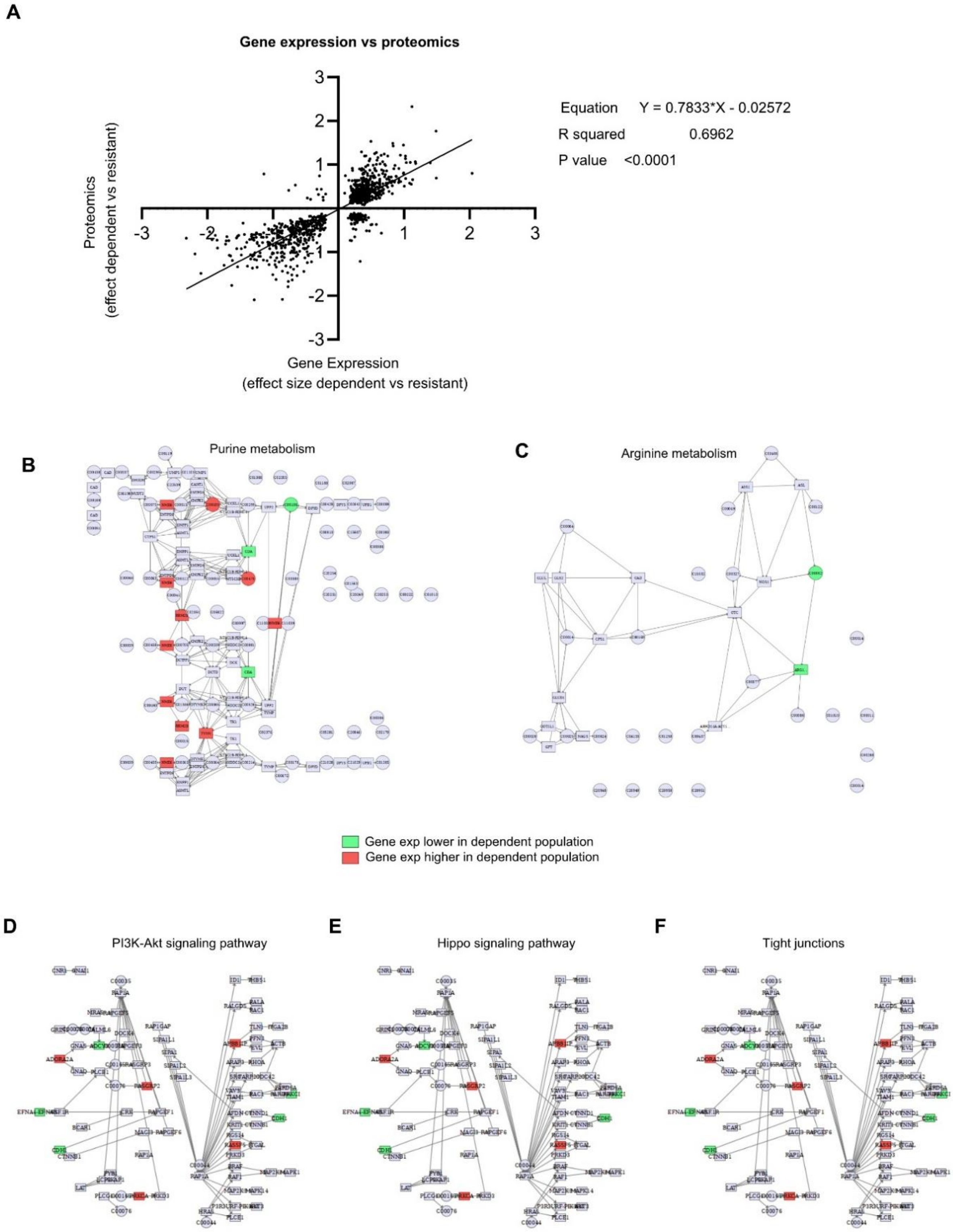

Figure S2. (A) X/Y plot showing concordance between DEGs from transcriptomic and proteomic datasets. Pathway maps for the top metabolic pathways (B-D), and signaling pathways (D–F) from Metaboanalyst joint pathway analysis.

Supplemental material related to Figure 3. Integrated gene dependency and drug sensitivity profiling identifies therapeutic vulnerabilities in HSP90AB1-resistant cells.

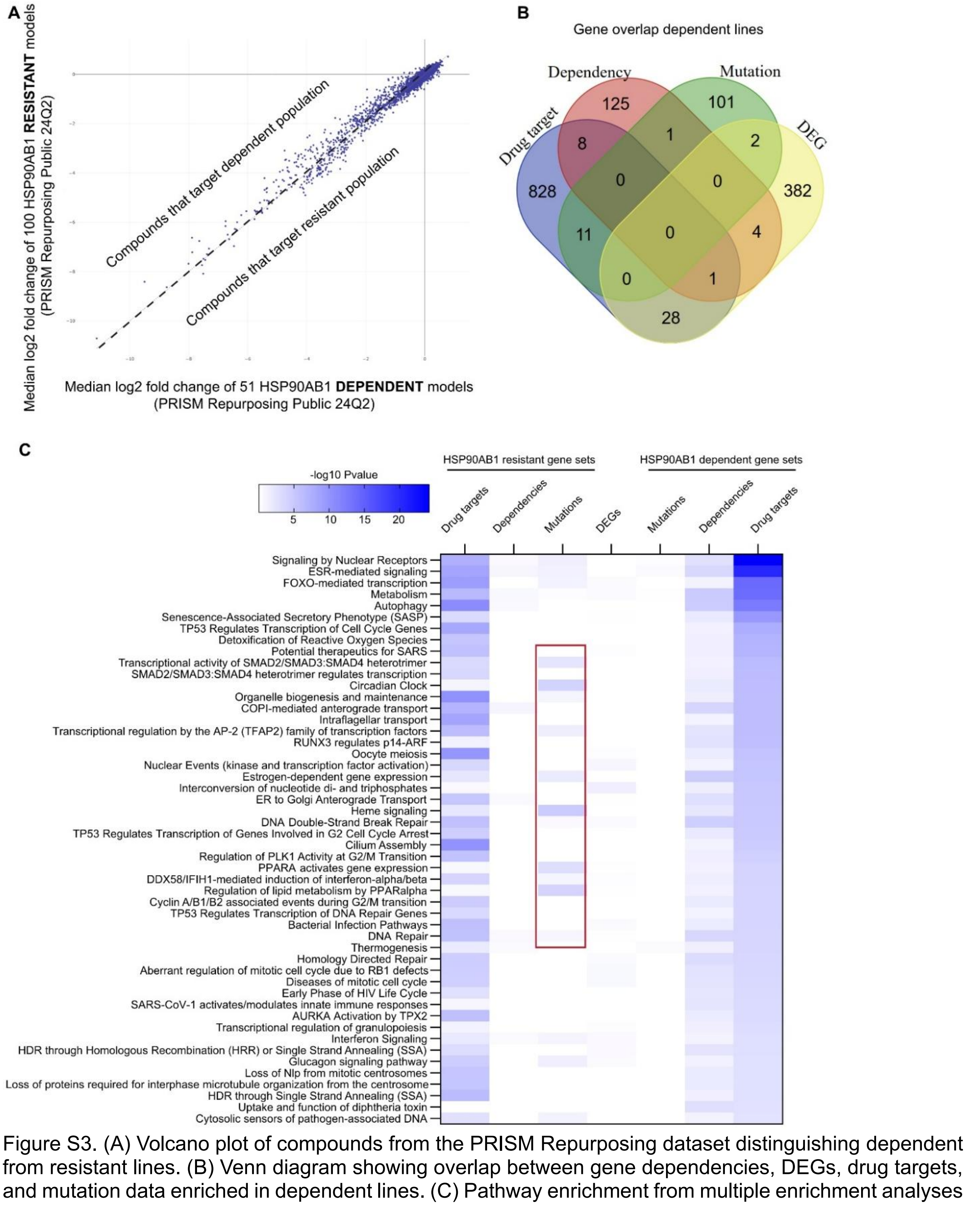

Figure S3. (A) Volcano plot of compounds from the PRISM Repurposing dataset distinguishing dependent from resistant lines. (B) Venn diagram showing overlap between gene dependencies, DEGs, drug targets, and mutation data enriched in dependent lines. (C) Pathway enrichment from multiple enrichment analyses (PANGEA), highlighting the top 50 pathways for the genes that are drug targets in HSP90AB1-dependent lines compared to parallel analyses in DEGs, dependencies and drug target gene lists.

Supplemental material related to Figure 4. Shared and unique molecular features of NDNB-25-sensitive and -resistant populations.

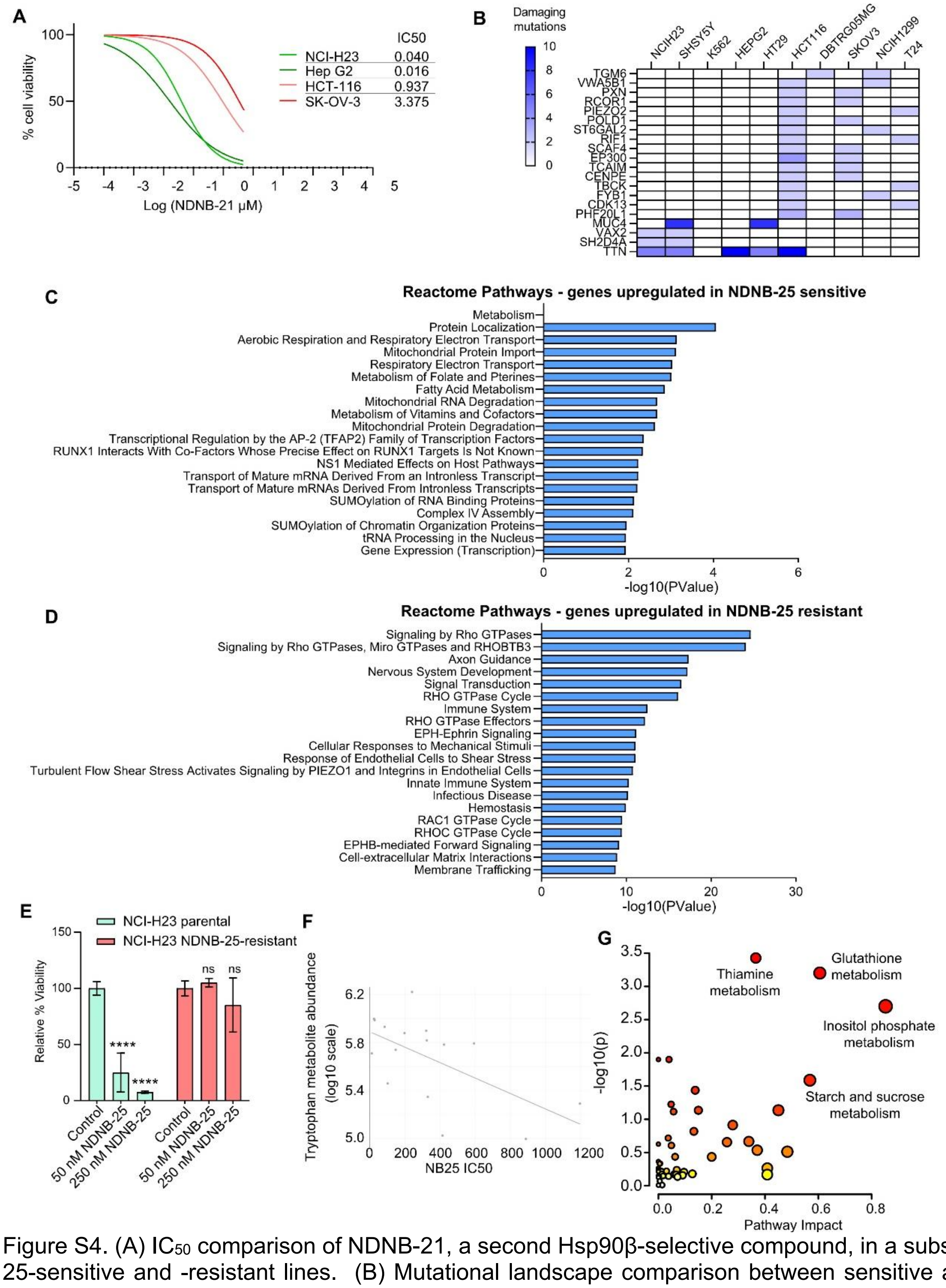

Figure S4. (A) IC_{50} comparison of NDNB-21, a second Hsp90β-selective compound, in a subset of NDNB-25-sensitive and -resistant lines. (B) Mutational landscape comparison between sensitive and resistant lines. (C–D) Enrichment analysis of Reactome pathways for DEGs upregulated in NDNB-25 sensitive (C) and NDNB-25 resistant lines (D). (E) Relative % Cell viability for NCI-H23 parental line and NDNB-25 acquired resistance line, treated with 50 or 250 nM NDNB-25. (F) X/Y plot of NDNB-25 sensitivity (IC_{50}) and metabolite abundance for tryptophan. (G) Joint pathway analysis of DEGs from gene expression and metabolites from metabolomics data.

Raw blots and densitometry related to Figure 4G

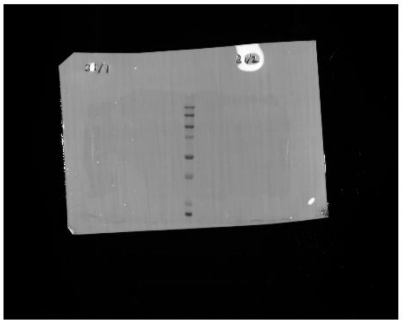

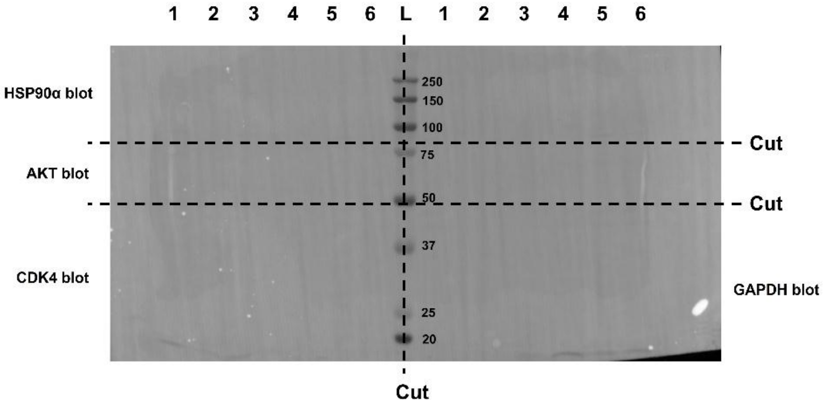

Cut membrane to see multiple targets with different molecular weights.
Each sample ran twice on same gel because 2 targets had similar molecular weight.

1 NCI-H23 Control
2 NCI-H23 100 nM NDNB-25
3 NCI-H23 1000 nM NDNB-25
4 NCI-H23 Resistant Control
5 NCI-H23 Resistant 100 nM NDNB-25
6 NCI-H23 Resistant 1000 nM NDNB-25
L Ladder
1 NCI-H23 Control
2 NCI-H23 100 nM NDNB-25
3 NCI-H23 1000 nM NDNB-25
4 NCI-H23 Resistant Control
5 NCI-H23 Resistant 100 nM NDNB-25
6 NCI-H23 Resistant 1000 nM NDNB-25

GAPDH

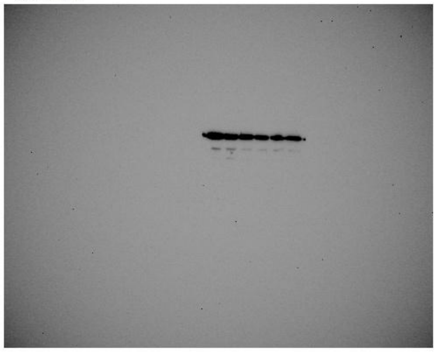

AKT

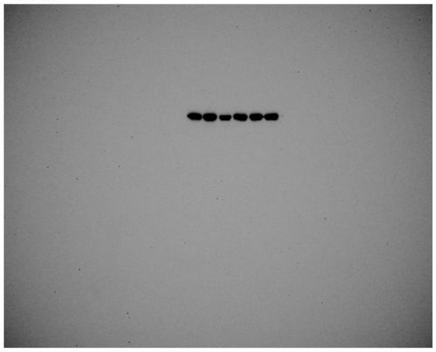

HSP90α

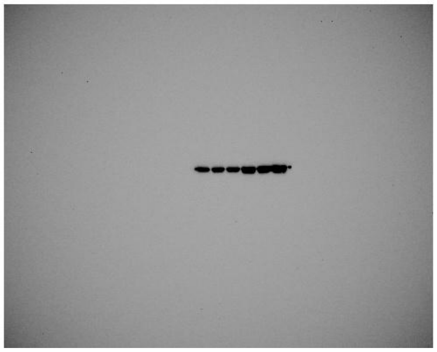

CDK4

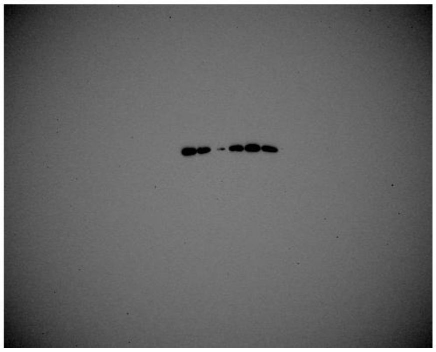

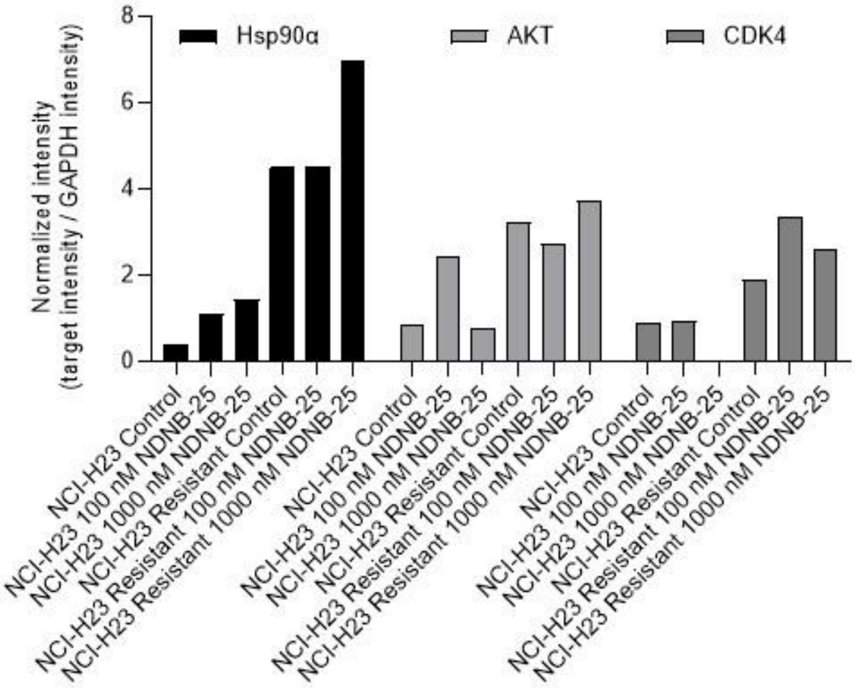

Lane No.	Sample	GAPDH Adj. Total Band Vol. (Int)	HSP90A Adj. Total Band Vol. (Int)	AKT Adj. Total Band Vol. (Int)	CDK4 Adj. Total Band Vol. (Int)
1	NCI-H23 Control	1458782	610650	1274460	1317630
2	NCI-H23 100 nM NDNB-25	606398	661726	1469431	577716
3	NCI-H23 1000 nM NDNB-25	464438	671785	370272	0
4	NCI-H23 Resistant Control	313118	1412677	1009606	591948
5	NCI-H23 Resistant 100 nM NDNB-25	371436	1676046	1018133	1241364
6	NCI-H23 Resistant 1000 nM NDNB-25	249002	1735680	927630	645510

Supplemental material related to Figure 5. Comparative analysis of HSP90AB1 gene dependency and NDNB-25 sensitivity reveals unique co-dependencies and drug sensitivities.

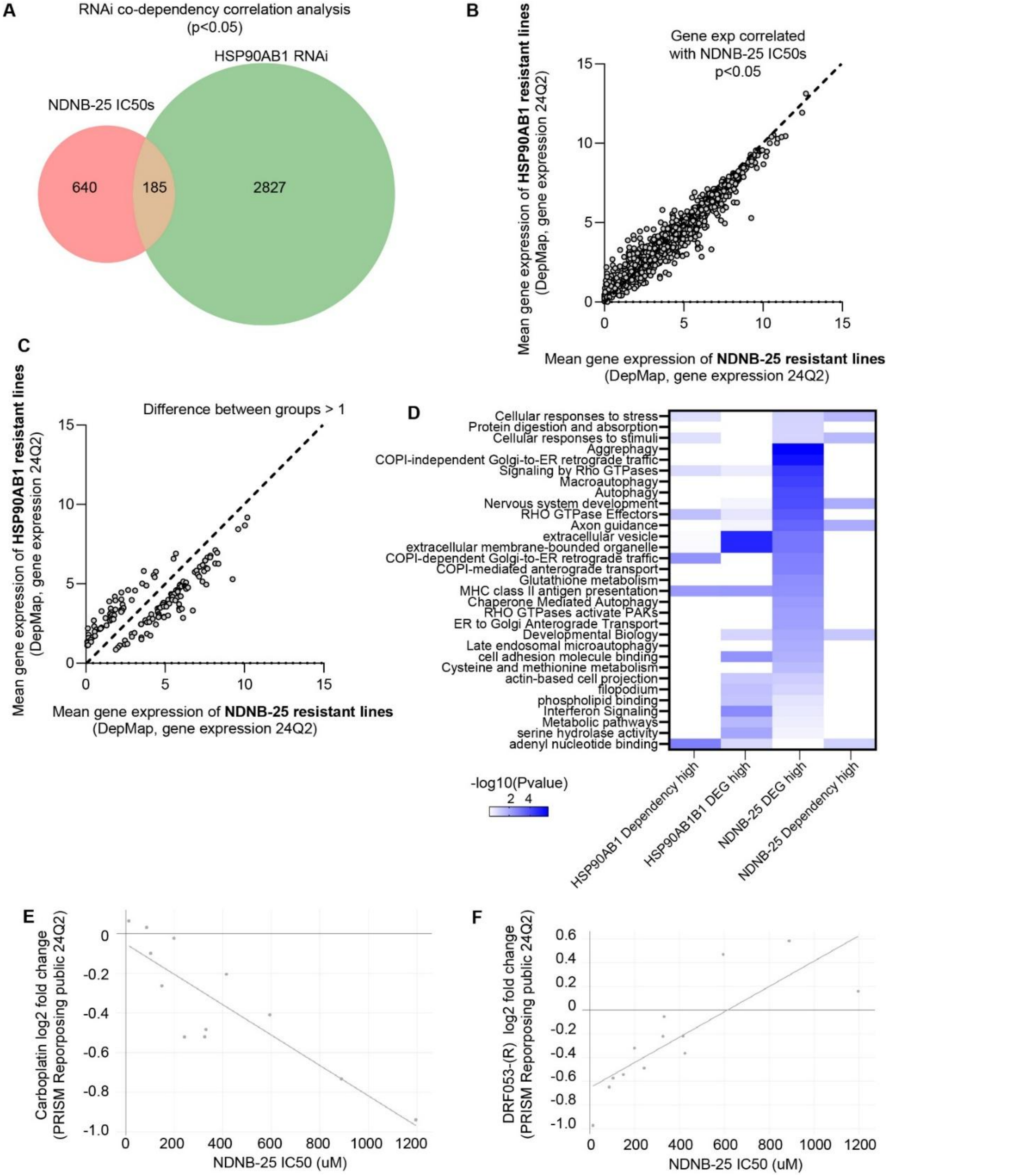

Figure S5. (A) Comparison of co-dependencies related to drug-sensitive (NDNB-25 IC_{50} values) and gene dependent (HSP90AB1 RNAi) co-dependency correlation analyses. (B–C) X/Y plots showing differential gene expression between NDNB-25- and HSP90AB1-resistant lines. (D) Enrichment analysis of DEGs and dependencies unique to NDNB-25 resistance. (E–F) Representative compound correlation plots showing positive and negative correlations with NDNB-25 IC_{50}s.

Supplemental material related to Figure 6. Integrated network analysis and functional validation of therapeutic vulnerabilities in NDNB-25-resistant cells.

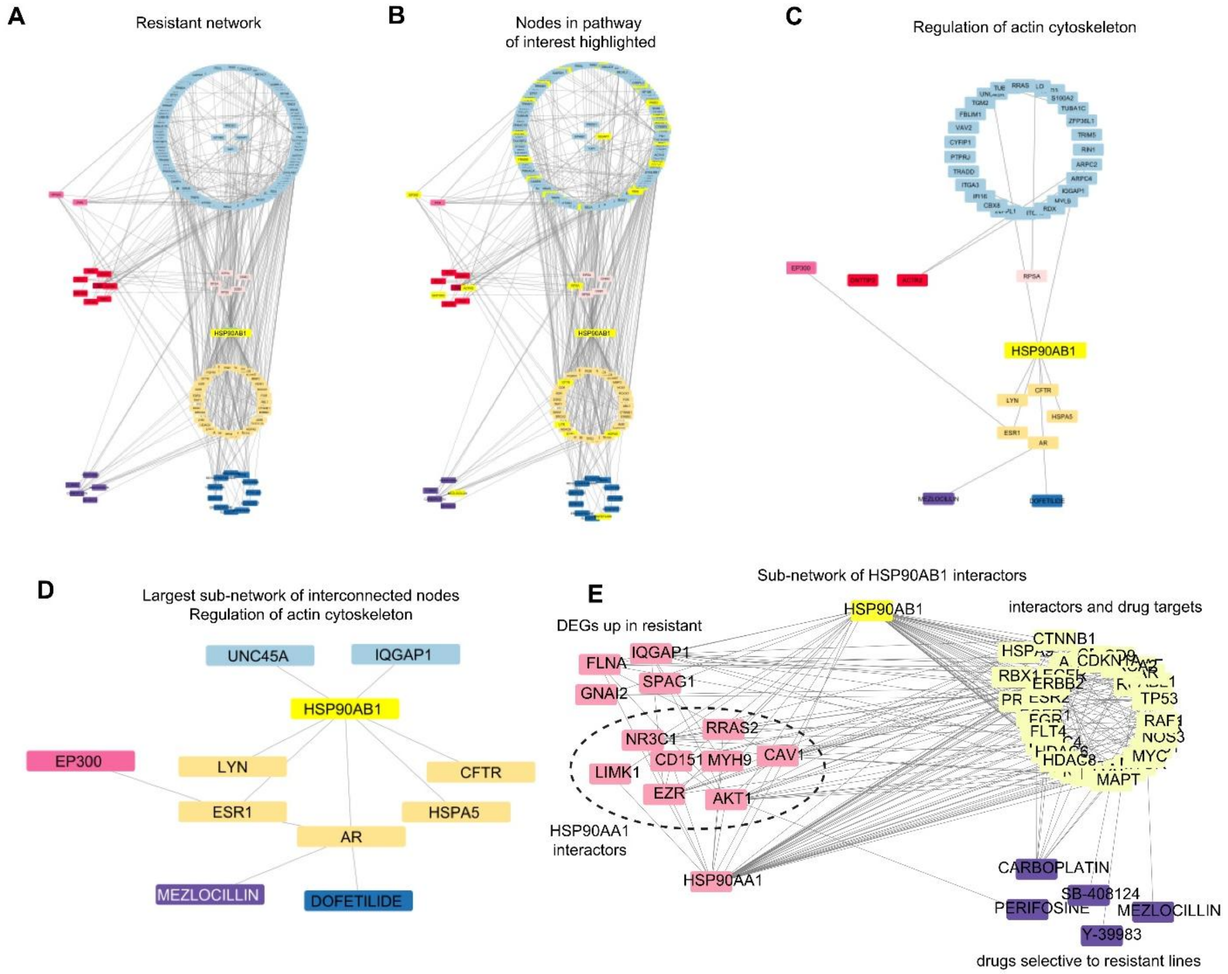

Figure S6. (A-D) Workflow used in generating integrated gene–drug–dependency networks for visualization and data exploration and extracting functional modules. Identification of subnetworks based on pathway enrichment terms, illustrated using the actin cytoskeleton regulation pathway as an example (B and C). Focused subnetwork of genes involved in actin cytoskeleton regulation, containing DEGs, Hsp90β interactors, and drug targets elevated in NDNB-25-resistant lines (D). (E) Largest subnetwork of Hsp90β interactors, showing connections between relevant drugs that target resistant-lines, and Hsp90α interactors.
